# Reinforcement feedback impairs locomotor adaptation and retention

**DOI:** 10.3389/fnbeh.2024.1388495

**Published:** 2024-04-24

**Authors:** Christopher M. Hill, Emerson Sebastião, Leo Barzi, Matt Wilson, Tyler Wood

**Affiliations:** ^1^Department of Kinesiology and Physical Education, Northern Illinois University, Dekalb, IL, United States; ^2^School of Kinesiology, Louisiana State University, Baton Rouge, LA, United States; ^3^Department of Health and Kinesiology, University of Illinois Urbana-Champaign, Urbana, IL, United States; ^4^School of Allied Health and Communicative Disorders, Northern Illinois University, Dekalb, IL, United States

**Keywords:** reinforcement, reward, punishment, locomotor adaptation, supervised learning, feedback, motor memory

## Abstract

**Introduction:**

Locomotor adaptation is a motor learning process used to alter spatiotemporal elements of walking that are driven by prediction errors, a discrepancy between the expected and actual outcomes of our actions. Sensory and reward prediction errors are two different types of prediction errors that can facilitate locomotor adaptation. Reward and punishment feedback generate reward prediction errors but have demonstrated mixed effects on upper extremity motor learning, with punishment enhancing adaptation, and reward supporting motor memory. However, an in-depth behavioral analysis of these distinct forms of feedback is sparse in locomotor tasks.

**Methods:**

For this study, three groups of healthy young adults were divided into distinct feedback groups [Supervised, Reward, Punishment] and performed a novel locomotor adaptation task where each participant adapted their knee flexion to 30 degrees greater than baseline, guided by visual supervised or reinforcement feedback (Adaptation). Participants were then asked to recall the new walking pattern without feedback (Retention) and after a washout period with feedback restored (Savings).

**Results:**

We found that all groups learned the adaptation task with external feedback. However, contrary to our initial hypothesis, enhancing sensory feedback with a visual representation of the knee angle (Supervised) accelerated the rate of learning and short-term retention in comparison to monetary reinforcement feedback. Reward and Punishment displayed similar rates of adaptation, short-term retention, and savings, suggesting both types of reinforcement feedback work similarly in locomotor adaptation. Moreover, all feedback enhanced the aftereffect of locomotor task indicating changes to implicit learning.

**Discussion:**

These results demonstrate the multi-faceted nature of reinforcement feedback on locomotor adaptation and demonstrate the possible different neural substrates that underly reward and sensory prediction errors during different motor tasks.

## Introduction

Adaptation is fundamental to the human motor behavior system, where motor commands must be constantly updated and remain flexible to meet environmental and task demands (Wei and Körding, 2009). Motor adaptation requires the gradual reduction of errors by reducing the discrepancy of predicted and actual sensory feedback [sensory prediction error (SPE)] (Shadmehr et al., 2010; Izawa and Shadmehr, 2011; Krakauer et al., 2019). Resolving these prediction errors adjusts the internal model of movement in the cerebellum, resulting in lower error and better performance as the task progresses (Baumann et al., 2015). Motor adaptation can also be achieved by maximizing rewards or avoiding punishments [reward prediction error (RPE)] and relies on a different neural source of error computation and correction (Jocham and Ullsperger, 2009; Diederen et al., 2017). Interestingly, reward and punishment have demonstrated differential effects on motor adaptation and retention. For instance, during upper extremity reaching tasks punishment feedback enhances learning while reward facilitates increased task retention (Galea et al., 2015; Song and Smiley-Oyen, 2017; Hamel et al., 2018; Hill et al., 2020, 2021; Yin et al., 2023). Moreover, these two forms of feedback facilitate motor adaptation via different neural pathways (Wrase et al., 2007; Hester et al., 2010; Galea et al., 2011; Spampinato et al., 2019; Hill et al., 2020, 2021; Spampinato and Celnik, 2021).

Locomotor adaptation is a motor learning process used to alter either the spatial and/or temporal elements of walking and has received considerable interest in the previous decades as a method of optimizing neurorehabilitation (Roemmich and Bastian, 2018; Hinton et al., 2020; Dzewaltowski et al., 2021; Severini and Zych, 2022). Multiple methods have been developed to elicit adaptation during walking, such as using asymmetrically moving treadmill belts, which alters the kinematic relationship between the lower extremities (Reisman et al., 2005; Morton and Bastian, 2006; Reisman et al., 2010; Torres-Oviedo et al., 2011; Malone et al., 2012; Long et al., 2016; Gonzalez-Rubio et al., 2019; Hinton et al., 2020; Buurke et al., 2022; Severini and Zych, 2022). Others have utilized augmented feedback to modify joint angular kinematics or step length (Roemmich and Bastian, 2015; Roemmich et al., 2016; Statton et al., 2016; Cherry-Allen et al., 2018; Leech and Roemmich, 2018). Adaptation during these paradigms relies primarily on the cerebellum to resolve SPEs and update the internal model of the locomotor behavior (Morton and Bastian, 2006; Jayaram et al., 2012; Hoogkamer et al., 2015; Hinton et al., 2020).

Previous investigations have leveraged reinforcement feedback during locomotor adaptation paradigms (Hasson et al., 2015; Sato et al., 2022). These studies have found contrasting results to those provided during upper extremity reaching tasks. For instance, categorical reinforcement during a gait adaptation task did not provide any added benefit to adaptation or immediate retention (Hasson et al., 2015). Others have noted punishment can enhance locomotor savings and reward lowers adaptation rates compared to neutral visual feedback (Sato et al., 2022). These findings are unlike those found in the upper extremity and may suggest different neural pathways for the promotion of adaptation and retention between upper and lower extremities adaptation tasks. Hinton et al. (2020) discussed this possibility, as areas such as the primary motor cortex (M1) are more involved in locomotor adaptation rather than retention which is not the case in upper extremities (Izawa and Shadmehr, 2011; Galea et al., 2015; Quattrocchi et al., 2018; Spampinato et al., 2019; Hill et al., 2020, 2021; Spampinato and Celnik, 2021; Wu et al., 2022). These differences may stem from the differing goals in between the tasks. Unlike the discrete nature of reaching, walking is a mostly continuous skill, with significant differences in neural control, intralimb coordination, interlimb coordination, and energy optimization. In fact, there is limited generalization between upper and lower extremity tasks (Bakkum et al., 2020, 2021). These contrasting findings reveal the need for further exploration of reinforcement feedback during complex locomotion acquisition and retention.

This study’s purpose was to determine the effects of reinforcement feedback on adaptation, retention, and savings during a novel locomotor adaptation task. We hypothesized that supervised and reinforcement feedbacks will drive locomotor adaptation at similar rates, but reward will induce greater retention and savings compared to sensory and punishment feedback. To test this hypothesis, we developed a knee flexion angle adaptation task similar to that of previous studies using healthy neurological intact subjects (Statton et al., 2016) and persons with stroke (Cherry-Allen et al., 2018), and provided either visual or graded reinforcement feedback during a walking bout with different conditions to assess both the adaptation, retention, and savings. Our results suggest that reinforcement feedback (reward and punishment) is detrimental to the processes that induce fast locomotor adaptation and encoding robust motor memories.

## Methodology

### Participants

All procedures were approved by the Northern Illinois University Institutional Review Board [protocol number HS21-0399] and align to the principles expressed in the Declaration of Helsinki. All participants provided written informed consent prior to participating. Recruitment occurred between 15 June 2022 and 15 September 2022. Thirty-three young healthy adults participated in this study [age range: 19–34 years, mean age ± standard deviation (SD): 24.61 ± 3.97 years, body height: 170.66 ± 34.32 cm, body weight: 77.28 ± 15.57 kg, males: 16, females: 17]. Participants were classified as right-handed using the Edinburgh Handedness Inventory (EHI) (>50 = right-handed, mean handedness score ± SD: 94.74 ± 10.40) and were free of major physiological (musculoskeletal, neurological, cardiovascular) and psychological (drug abuse, depression, generalized anxiety) disorders. The derived sample size is based on an *a priori* power calculation conducted in G*power 3.1.9.4 software using the effect size of a previous study investigating behavioral effects of reinforcement feedback on task error during walking (Sato et al., 2022). From this study, we calculated an effect size (Cohen’s *f*) of 0.47 and using *α* = 0.05 and *β* = 0.8. Participants were recruited from the local population of the University and the surrounding communities using word of mouth, electronic announcements, and posted flyers. Each participant was randomly allocated to one of three feedback groups [Reward (*n* = 11), Punishment (*n* = 11), Supervised (*n* = 11)]. Descriptive data of each group can be found in Table 1. The Behavioral Avoidance/Inhibition scales (BAS/BIS) were used to score sensitivity to reinforcement which is divided into four subcomponents (BAS FUN, BAS DRIVE, BAS REWARD RESPONSIVENESS, BIS). Additional information regarding these scales is available elsewhere (Aluja and Blanch, 2011).

**Table 1:** Participant descriptive data.

Group	EHI	Age (Years)	Height (cm)	Weight (kg)	Leg Length (cm)	Step Length (m)	Treadmill Speed (m/s)
REWARD	100 ± 0.00	24.36 ± 4.22	170.95 ± 12.77	80.49 ± 19.09	80.91 ± 6.04	0.53 ± 0.04	0.71 ± 0.05
PUNISHMENT	90.00 ± 14.14	24.36 ± 4.50	169.63 ± 8.67	76.52 ± 10.69	80.63 ± 5.92	0.53 ± 0.03	0.71 ± 0.05
SUPERVISED	94.23 ± 9.54	25.09 ± 3.47	171.41 ± 10.07	75.28 ± 16.84	81.81 ± 3.99	0.55 ± 0.02	0.72 ± 0.03

Presented as mean ± standard error.

### Data collection

**Figure 1:**
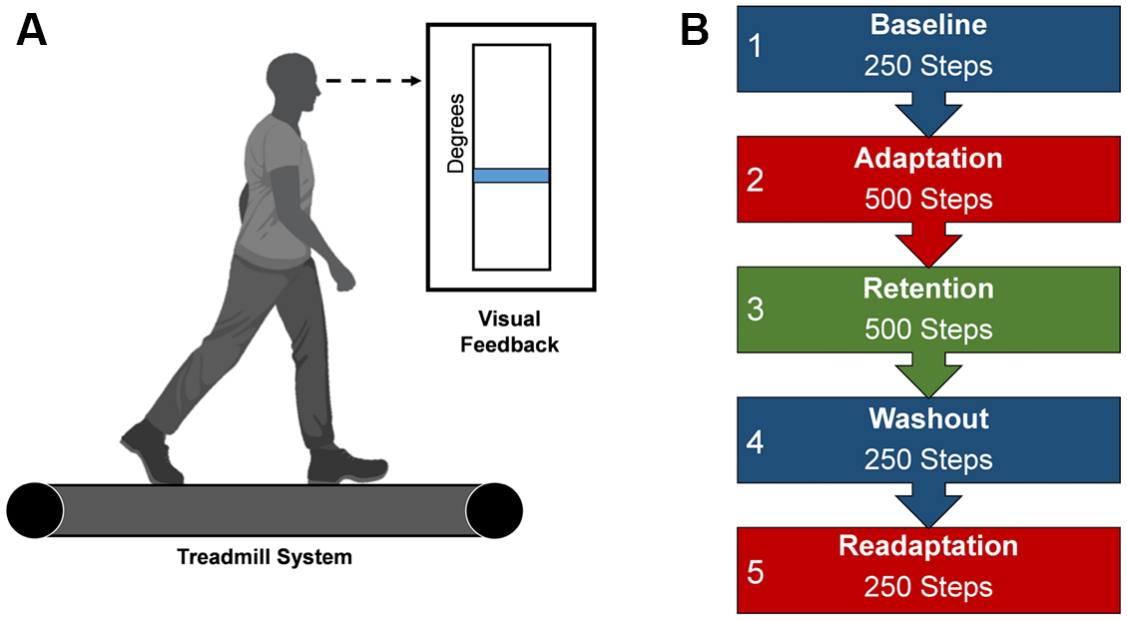
Locomotor learning task. **(A)** Visual feedback presented on a screen during the locomotor task. Blue line represents the current knee flexion angle, a mean of the previous two stride cycles. This blue line would change in height based as participants increased or decreased their knee joint flexion **(B)** Progression of task conditions. Duration is dictated by number steps. Created with BioRender.com.

Each participant adapted to a new walking pattern on a treadmill (Woodway, Waukesha, WI) with a different type of visual feedback in five distinct task conditions. All participants were informed of the nature of the task and the task goals by being read aloud a script before the start of the experiment, after which participants were asked to recall the task goals and the meaning of their assigned feedback to ensure understanding of the procedures. Questions from the participants concerning procedures and goals were addressed by the experimenters. To begin the protocol, participants began walking on the treadmill in a dimly lit room, to minimize external distractions and to increase the saliency of the presented feedback (Reisman et al., 2005), at a calculated treadmill speed based on each participant’s step length (two-thirds of the leg length (m)) multiplied by a cadence constant of 1.33 (90 steps/60 s) (Baseline). Leg length was determined as the distance from the greater trochanter to the lateral malleolus for each leg, and then averaged across both limbs. This method of calculating treadmill speed accounts for different heights among individuals and has been used in other studies using a similar population as in this study (Choi et al., 2016; Sato et al., 2022). The calculated speed of the treadmill was held constant throughout the experiment. Participants were instructed to walk as normally as possible while looking at the mounted screen in front of them. Lower limb angular kinematics of the segment of the lower right extremity was acquired by using two XSENS Mti-2 inertial measurement units (IMUs) affixed to the participant’s thigh and shank. IMU data was streamed into MATLAB (Natick, MA, United States) and custom script derived the participant’s knee flexion angle from the difference in the IMUs angular positions in reference to a world coordinate system (Baranek, 2012; Doan and Pham, 2019). Subsequently, participants were then provided real-time visual feedback in the form of blue line which numerically corresponded to their knee flexion angle during walking. This blue line would change in height based as participants increased or decreased their knee joint flexion (Figure 1A). After this steady-state walking bout, participants were visually cued with either visual feedback or reinforcement feedback to learn a new walking pattern by adapting their right knee’s movement during the swing phase to match a desired knee angle (Adaptation). The desired knee flexion angle was calculated as a 30-degree increase in the mean knee flexion angle during Baseline walking (Statton et al., 2016; Cherry-Allen et al., 2018). The task is a modified version of what was completed in Statton et al. (2016) and Cherry-Allen et al. (2018). Next, an errorless immediate retention phase occurred via removal of the group assigned feedback and replaced with null uninformative feedback (Retention). A washout period instructed participants to begin walking normally to return behavior to baseline levels (Washout). Subsequently, participants reengaged the adaptation task with the group feedback restored, to quantify task savings (Readaptation). All conditions were continuously collected and each participant will progressively go through each task condition with the number of steps dictate the duration (Figure 1B).

Visual feedback was displayed on a 60 cm screen, 85 cm in front of the participant, after every two steps with the right leg, completing a stride cycle. Adaptation and Readaptation used one of three feedbacks based on group assignment. The Reward and Punishment groups used a monetary scoring feedback system based on the difference between current and desired knee flexion angle. Participants were shown a number corresponding to a monetary gain or loss. The magnitude of scoring feedback was dependent on the amount of error in the previous stride cycle and followed these criteria:

- Reward: +4 points: meets desired angle; +3 points: within 10°; +2 points: within 20°; +1 point: within 30°; 0 points: exceeds or fewer than 30°.
- Punishment: 0 points: meets desired angle; −1 point: within 10°; −2 points: within 20°; −3 points: within 30°; −4 points: exceeds or fewer than 30°.

**Figure 2:**
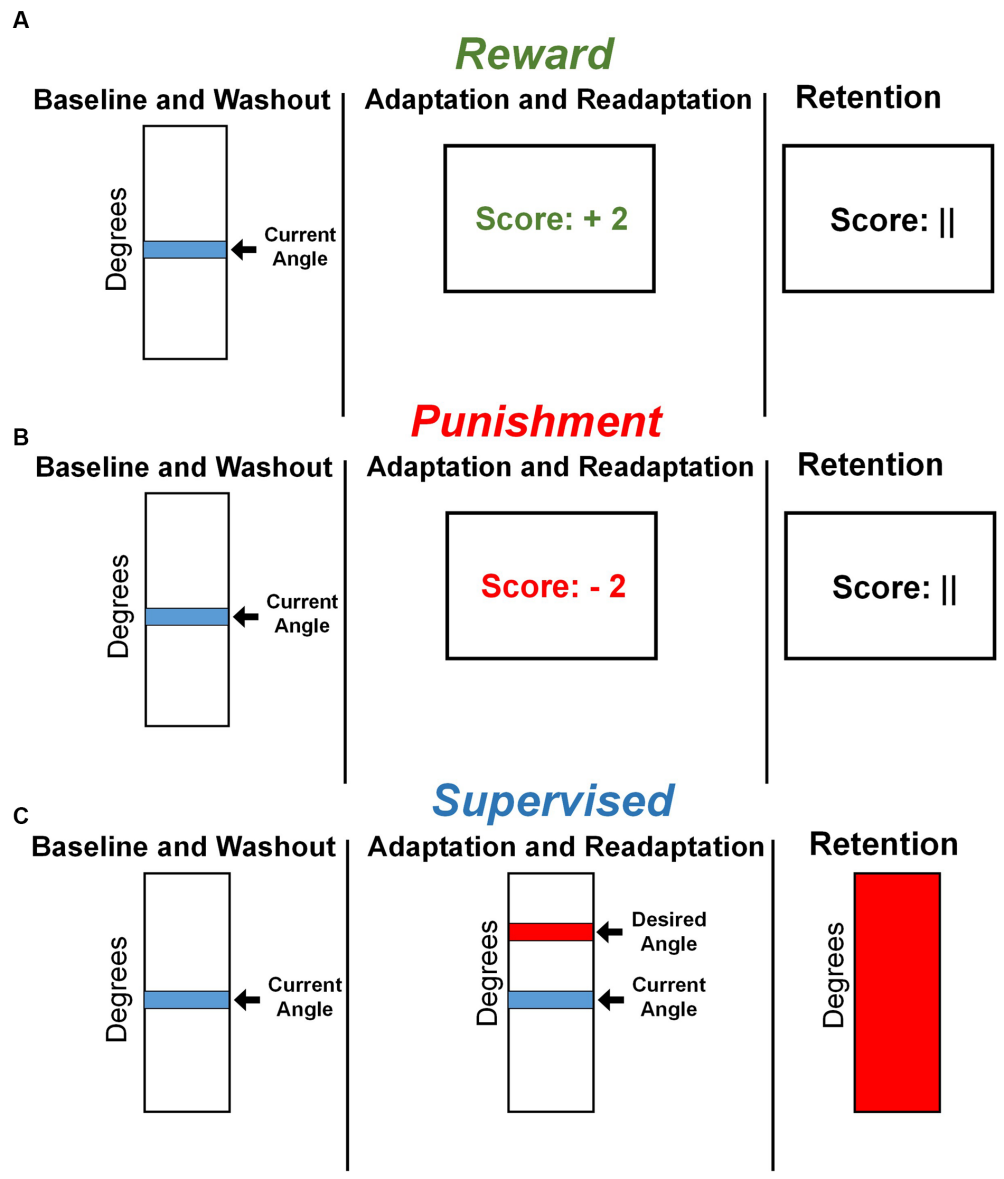
Feedback presentation by group. **(A)** Representative feedback during all task conditions for the Reward group. **(B)** Representative feedback during all task conditions for the Punishment group. **(C)** Representative feedback during all task conditions for the Supervised group.

All groups started with a total of zero points. Those in the Reward group earned positive points (Figure 2A), while those in the Punishment group accrued negative points (Figure 2B). The Reward group was instructed that they begin with USD 0.00 and earn money based on their performance. The Punishment group was instructed that they began with USD 30.00 and lost money based on their performance. Additionally, participants in the Supervised group were given the instructions of either the Reward or Punishment groups, to control the effects of the script. The Supervised group were provided a vertical scale with their current and target line representing a desired peak knee flexion angle. This target line remained in the same location throughout the experiment (Figure 2C). To ensure equity in compensation, all participants were compensated, despite group assignments, with the full USD 30.00 at the end of the study, regardless of performance.

The task goal was to minimize error by matching the current knee flexion angle with the desired knee flexion angle. The 30-degree increase was chosen to allow for a wider exploration of the task space. Previous studies have outlined that a wider task space facilitates greater rates of adaptation with reinforcement, by allowing participants to freely explore multiple movements to find the optimal action that meets task demands (Nikooyan and Ahmed, 2015; Cashaback et al., 2019). Retention featured null feedback in the form a filled vertical scale (Supervised) or null scoring feedback (Reward and Punishment). Feedback presentation followed the same timing latency, ten milliseconds after completing a stride cycle.

### Data analysis

Task error magnitude was calculated during Adaptation, Retention, and Readaptation. Task error is defined as the difference between the current and desired knee flexion angle relative to the mean baseline knee flexion angle. Adaptation, retention, and savings patterns were characterized by averaging task error magnitude values in 7 task epochs: Early Adaptation (first 10 steps of Adaptation), Mid-Adaptation (mean error between steps 100–200), Adaptation Plateau (last 100 steps of Adaptation), Early Retention (first 10 steps of Retention), Retention Plateau (last 100 steps of Retention) Early Readaptation (first 10 steps of Readaptation), Readaptation Plateau (last 100 steps of Readaptation) (Statton et al., 2016; Cherry-Allen et al., 2018). The analysis is a modified version of what was completed in Statton et al. (2016) and Cherry-Allen et al. (2018). To quantify the amount of implicit adaptation resulting from the protocol, movement aftereffects were examined as the difference between in baseline-corrected peak flexion angle during the Washout and Baseline walking conditions. Aftereffects are defined as the persistence of a motor behavior when the perturbation is removed and reflects a change in the internal representation of a movement.

### Statistical analysis

Participant descriptive data and BAS/BIS subscales were evaluated with separate One-way ANOVAs (analysis of variances) to determine differences between the three feedback groups (Supervised, Reward, Punishment). Task error magnitude was compared across feedback groups (Supervised, Reward, Punishment), and conditions (Adaptation, Retention, and Readaptation) using linear mixed models for repeated measures (Boisgontier and Cheval, 2016; Giboin et al., 2020; Kumari et al., 2020). Task error magnitude was held as the dependent variable, Feedback Group (Reward, Punishment, Supervised) and Task Epoch (Early Adaptation, Adaptation Plateau, Early Retention, Retention Plateau, Early Readaptation, Readaptation Plateau) were held as fixed effects and individual subjects were held as random factors. Differences in locomotor adaptation were assessed by comparing task error magnitude Early Adaptation and Adaptation Plateau. Changes to locomotor memory were assessed by comparing Adaptation Plateau, Early Retention, and Retention Plateau task error magnitude. Locomotor savings were assessed by comparing Early Adaptation, Early Readaptation, and Readaptation Plateau task error magnitude. Aftereffect was accessed with linear mixed models for repeated measures with baseline-corrected peak knee flexion angle held as the dependent variable, Feedback Group (Reward, Punishment, Supervised) and Task Epoch (Baseline, Washout) were held as fixed effects and individual subjects were held as random factors. The advantages associated with linear mixed models, as opposed to conventional statistical methodologies, encompass the capability to account for measurements nested within individual subjects, the accommodation of missing and unbalanced data, prevention of information loss attributable to data averaging, and the facilitation of enhanced parameter estimation through the implementation of a partial pooling strategy (Boisgontier and Cheval, 2016; Giboin et al., 2020; Kumari et al., 2020). Differences in Mid-adaptation task error magnitude was compared using a One-way ANOVA (analysis of variances) to determine differences between Reward, Punishment, and Supervised groups. *p*-values less than 0.05 were considered statistically significant. All follow-up analysis for main effects and interactions were performed with a *Sidak* correction. All statistical analysis was conducted in SPSS v26 (IBM Corp., Armonk, NY, United States).

## Results

### Participant characteristics

All groups demonstrated similar descriptive characteristics. A One-way ANOVA revealed there was no significant differences among the three testing groups for EHI (*F*(2, 30) = 2.857, *p* = 0.073), Age (*F*(2, 30) = 0.116, *p* = 0.891), Height (*F*(2, 30) = 0.082, *p* = 0.921), Body Weight (*F*(2, 30) = 0.320, *p* = 0.729), and Step Length (*F*(2, 30) = 0.144, *p* = 0.866). Moreover, all participants walked with similar treadmill speeds during the task (*F*(2, 30) = 0.144, *p* = 0.866). All groups demonstrated similar sensitivities to reinforcement feedback (Table 2). A One-way ANOVA revealed there was no significant difference among the three testing groups on the four subscales within the BAS/BIS scale [BAS Drive *p* = 0.354, BAS FUN *p* = 0.419, BAS Reward Responsiveness *p* = 0.823, BIS *p* = 0.896].

**Table 2:** BAS/BIS scores.

Group	BAS Drive	BAS FUN	BAS Reward	BIS
Reward	12.60 ± 0.50	12.30 ± 0.54	17.90 ± 0.57	21.30 ± 1.51
Punishment	11.64 ± 0.67	12.09 ± 0.53	17.45 ± 0.77	21.27 ± 1.30
Supervised	13.09 ± 0.48	11.55 ± 0.39	17.73 ± 0.51	20.18 ± 1.03

Presented as mean ± standard error.

## Locomotor performance

### Adaptation

**Figure 3:**
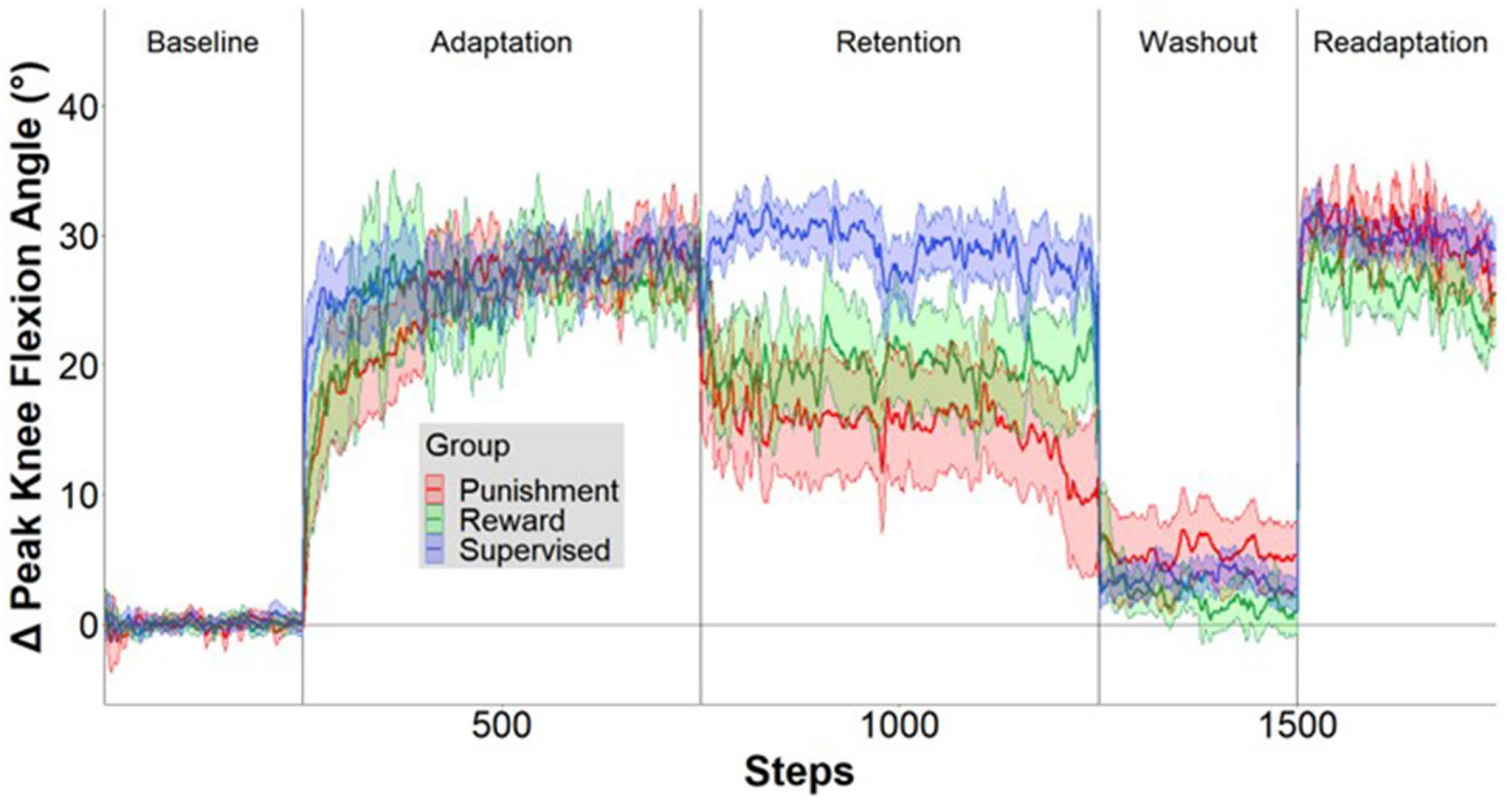
Locomotor performance time course. Mean ± standard error (SE) change in knee flexion angle over the time course of the five conditions relative to mean baseline walking for the Supervised (blue), Reward (green), and Punishment (red) groups. Data are smoothed with a running average by 2 strides and expressed relative to baseline. Shaded areas around each line represent SE.

**Figure 4:**
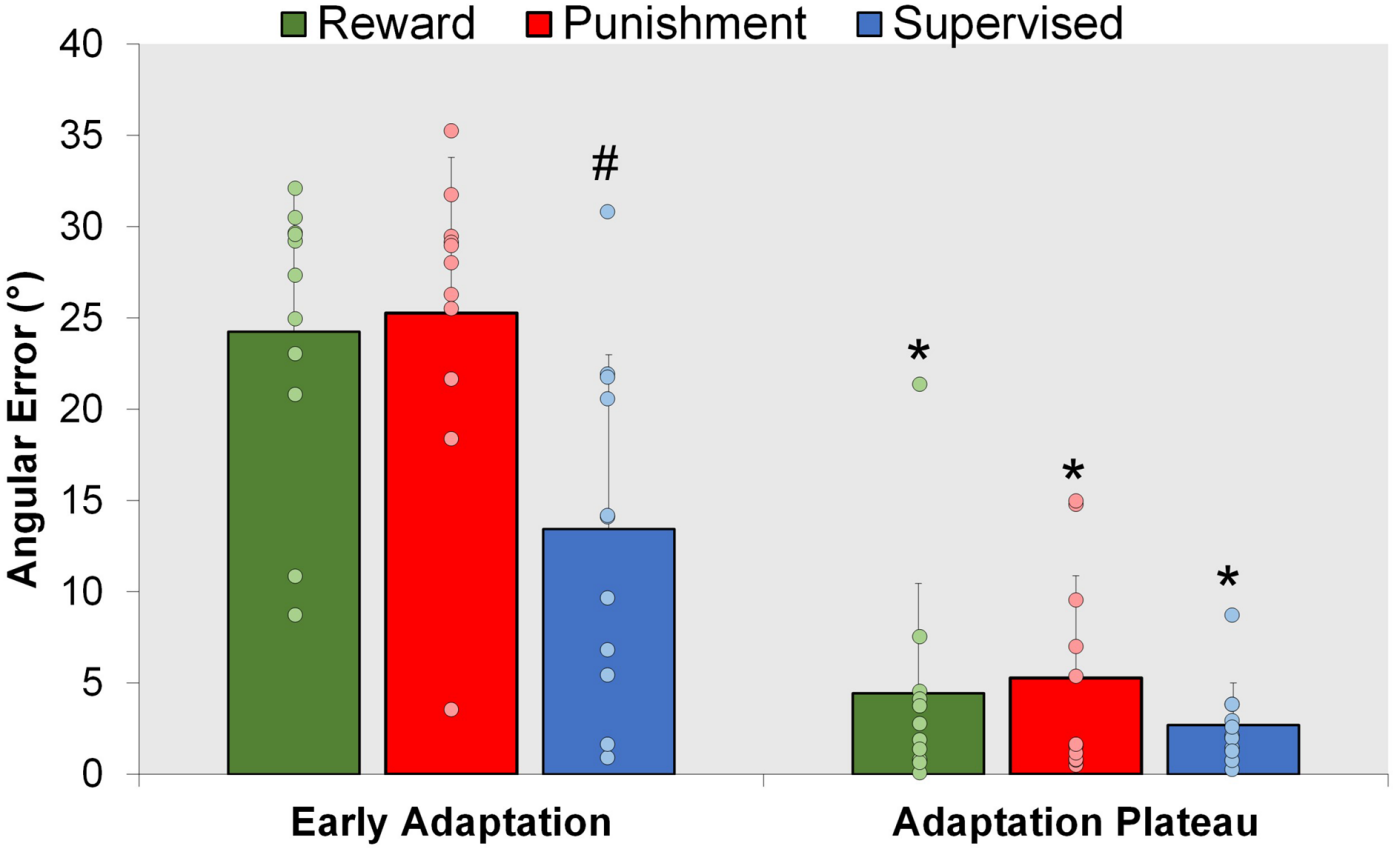
Adaptation. Mean task error magnitude for all groups during the Adaptation (Early Adaptation and Adaptation Plateau) condition. ∗*p* < 0.05 compared to Early Adaptation. #*p* < 0.05 compared to Reward and Punishment. Bars represent mean and dots represent the individual responses.

A representation of the time course of locomotor performance is presented in Figure 3. During Adaptation, all groups gradually reduced error as the task progressed, but the feedback affected task performance differently in certain task epochs (Figure 4). The Supervised feedback group showed faster learning during Early Adaptation compared to both Reward and Punishment groups. A significant Group × Condition interaction (*F*(2,30) = 4.226, *p* = 0.024) was found where the Supervised group displayed lower error during Early Adaptation compared to Reward [mean difference (MD): 10.812, *p* = 0.002, 95% confidence intervals (CIs) = 3.396–18.227] and Punishment [MD: 11.829, *p* = 0.001, 95% CIs = 4.414–19.224] groups, indicating supervised feedback created faster adaptation than reinforcement. No significant differences were found between groups (*p* > 0.05) when examining the Adaptation Plateau.

However, all groups demonstrated a similar level of error 200 steps into Adaptation. No significant differences were noted between groups during Mid-adaptation (*F*(2,30) = 1.071, *p* = 0.356). Moreover, all groups learned as they progressed from Early Adaptation to the Adaptation Plateau. Significantly lower error was found during the Adaptation Plateau compared to Early Adaptation for Reward (MD: 19.804, *p* < 0.001, 95% CIs = 14.548–25.060), Punishment (MD: 19.980, *p* < 0.001, 95% CIs = 14.724–25.236) and Supervised (MD: 10.730, *p* < 0.001, 95% CIs = 5.474–15.986) groups. There were no significant differences found between groups during the Adaptation Plateau. Supervised feedback demonstrated similar error compared to Reward (MD: 1.738, *p* = 0.918, 95% CIs = −9.153-5.678) and Punishment (MD: 2.579, *p* = 0.779, 95% CIs = −9.995-4.836) feedback.

### Retention

**Figure 5:**
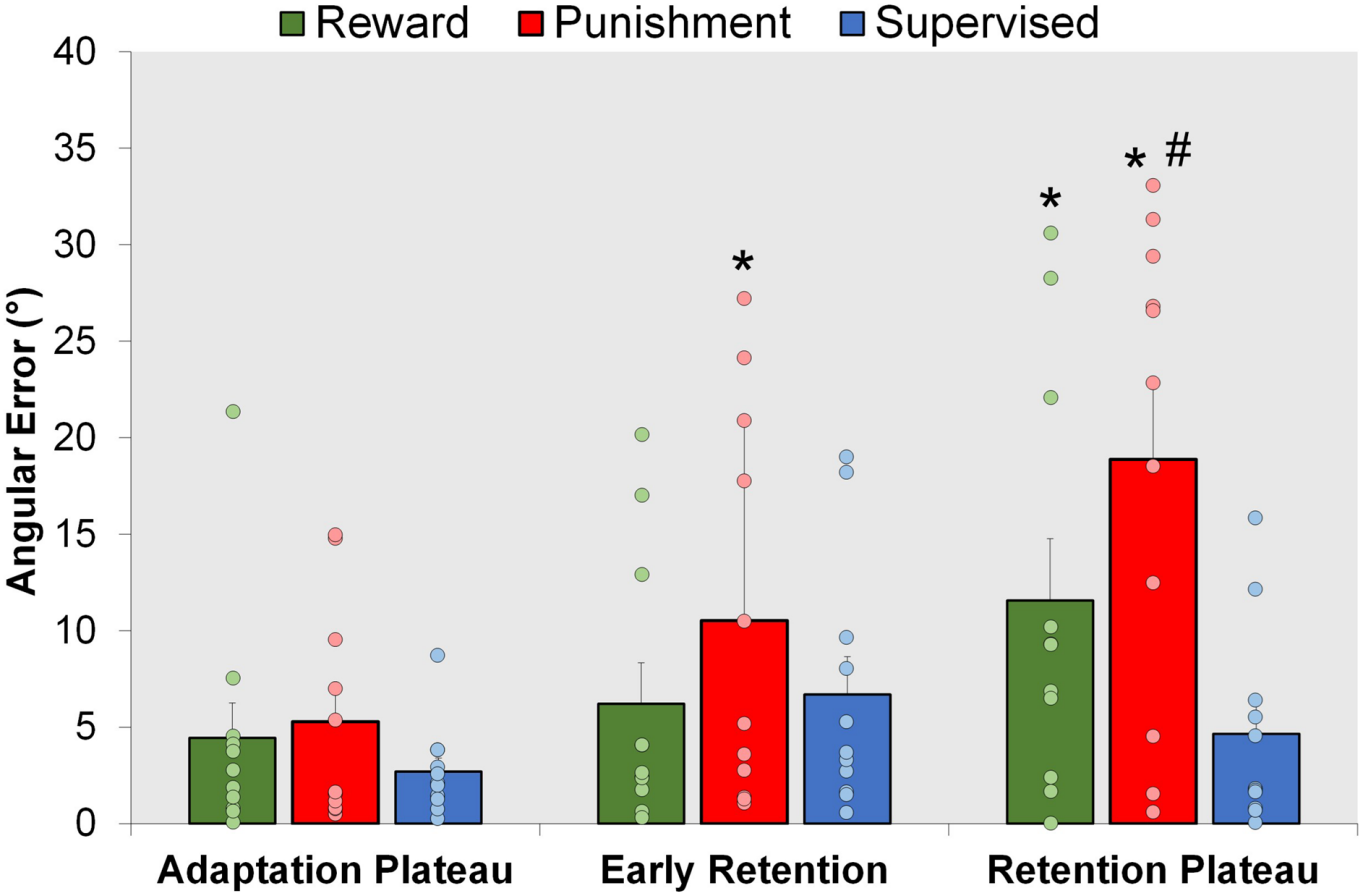
Retention. Mean task error magnitude for all groups during the Adaptation Plateau and the Retention (Early Retention and Retention Plateau) conditions. ∗*p* < 0.05 compared to Adaptation Plateau. #*p* < 0.05 compared to Control. Bars represent mean and dots represent the individual responses.

Punishment and Reward feedback displayed an increase in task error during the errorless retention period. Moreover, supervised feedback enhanced locomotor retention in comparison to Punishment, but was like Reward feedback (Figure 5). A significant Group × Condition interaction (*F*(4,60) = 3.623, *p* = 0.010) where an increased task error was found during Retention Plateau for the Punishment group in comparison to the Adaptation Plateau [MD: −13.598, *p* < 0.001, 95% CIs = 7.559–19.639] and Early Retention [MD: 8.358236, *p* = 0.004 95% CIs = 2.318–14.399]. Similarly, the Reward group demonstrated increased task error during the Retention Plateau in comparison to the Adaptation Plateau [MD: 7.121, *p* = 0.016, 95% CIs = 1.081–13.162]. Task error magnitude was similar across all conditions for the Supervised feedback group (*p* > 0.05). Upon closer examination of the group differences within conditions, all groups demonstrated a similar level of error during Early Retention (*p* > 0.05). However, during Retention Plateau, Punishment had an increased task error in comparison to supervised feedback [MD: 14.223, *p* < 0.001, 95% CIs = 6.0202–22.426] but not Reward [MD: 7.319, *p* = 0.094, 95% CIs = 0.883–15.522].

### Aftereffect

**Figure 6:**
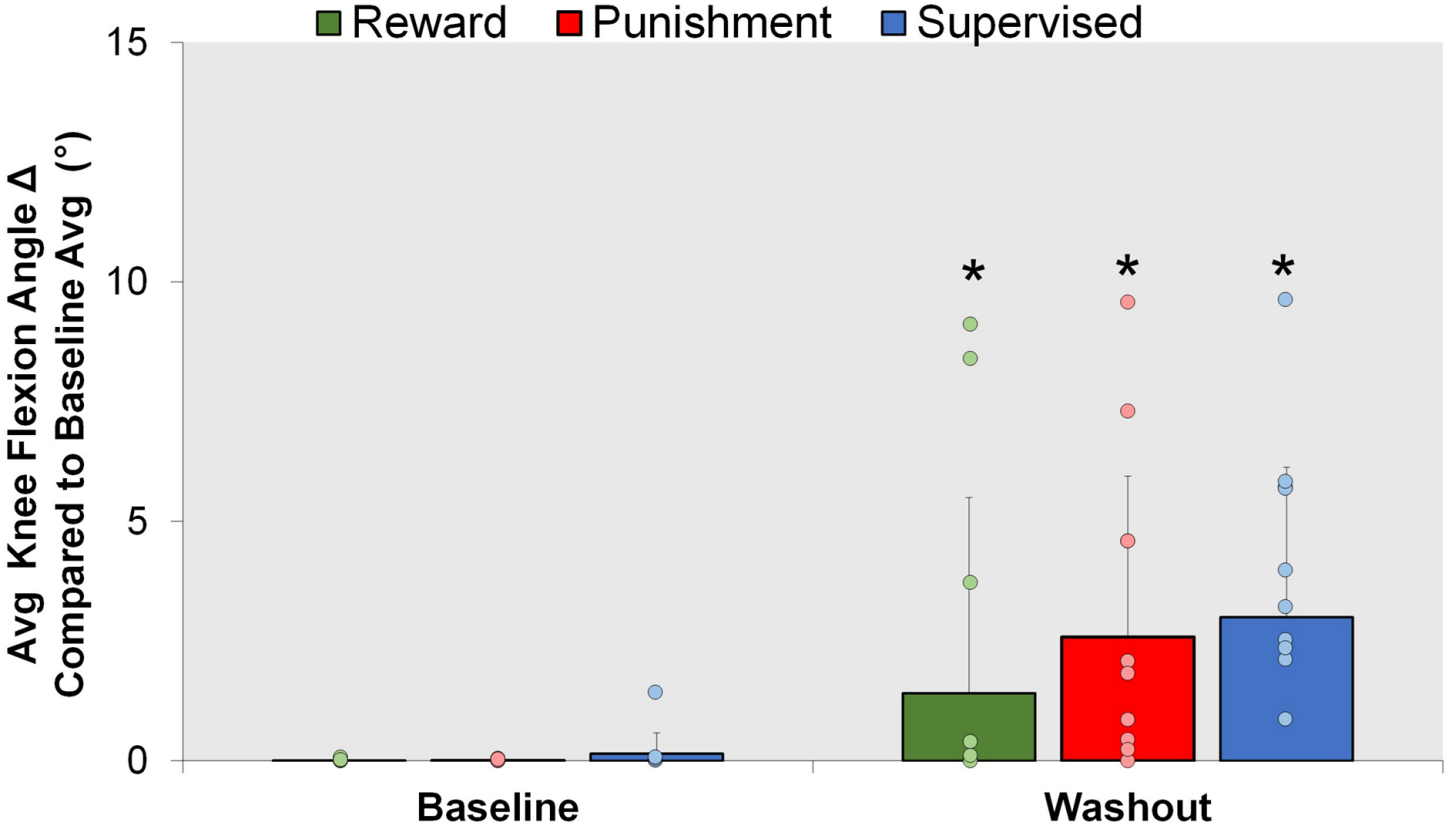
Aftereffect. Mean knee flexion angle for all groups during the Baseline and Washout conditions. ∗*p* < 0.05 compared to Baseline. Bars represent mean and dots represent the individual responses.

After the retention phase, all groups were told to return to normal walking; however, an increase in knee flexion was found during the Washout condition compared to Baseline (Figure 6). A significant main effect was found for condition (*F*(1,30) = 19.282, *p* < 0.001). Washout demonstrated a significantly higher knee flexion angle compared to Baseline [MD: 2.900, *p* < 0.001, 95% CIs = 1.551–4.249]. No significant interactions or main effects for group were found for the aftereffects (*p* > 0.05). These findings signify some adaptation is carried over as result of repeated performance and not mediated by feedback type.

### Savings

**Figure 7:**
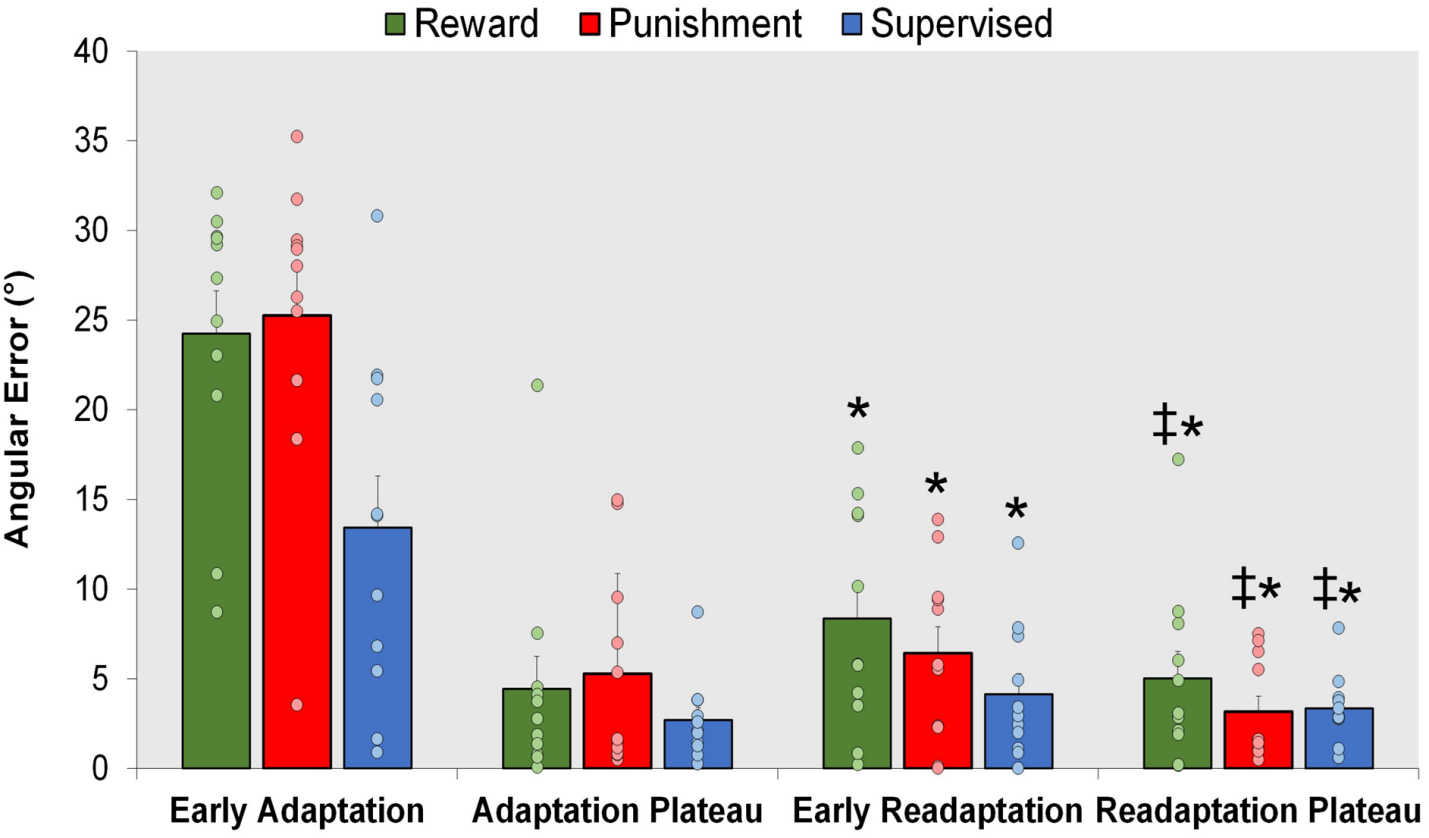
Savings. Mean task error magnitude for all groups during Early Adaptation, and Readaptation (Early Readaptation and Readaptation Plateau). ∗*p* < 0.05 compared to Early Adaptation. ‡*p* < 0.05 compared to Early Readaptation. Bars represent mean and dots represent the individual responses.

All groups exhibited locomotor savings, by having lower error upon re-exposure to the knee flexion adaptation task during Early Readaptation (Figure 7). However, there were no significant differences found between groups suggesting no effect of feedback type on locomotor savings. A significant main effect was found for Condition (*F*(1,30) = 102.027, *p* < 0.001) where Early Readaptation displayed lower error in comparison to Early Adaptation. No significant differences were observed between groups (*p* > 0.05). However, error magnitude decreased as the Readaptation conditioned progressed [MD: 14.669, *p* < 0.001, 95% CIs = 11.703–17.634]. A significant main effect was found for Condition (*F*(1,30) = 9.002, *p* = 0.005) where the Readaptation Plateau displayed a lower task error in comparison to Early Readaptation [MD: 2.461, *p* = 0.005, 95% CIs = 0.786–4.136]. No significant interactions or main effects for group were found during locomotor savings (*p* > 0.05).

## Discussion

This study sought to disassociate the roles of supervised and reinforcement feedback in a novel locomotor adaptation task. We found that the participants learned the adaptation task with all types of external feedback provided. However, contrary to our initial hypothesis, enhancing sensory feedback with a visual representation of the knee angle accelerated early adaptation and preserved the movement late into retention testing. In comparison, reinforcement feedback (i.e., reward and punishment) did not enhance the rate of adaptation, retention, and savings. Specifically, Reward and Punishment displayed lower early adaptation and Punishment diminished retention in comparison to supervised feedback. Moreover, all feedback enhanced the aftereffect of locomotor task indicating changes to implicit learning.

We found that enhancing sensory feedback by visually representing the participant’s knee flexion angle enhanced locomotor adaptation compared to reinforcement during the task’s early phases. Our findings confirm and contrast previous studies examining adaptation in the upper and lower extremities. First, our findings are like a previous study which demonstrated that providing supervised feedback of an error drives changes to locomotor behavior (Roemmich et al., 2016). This study provided a visual discrepancy of step lengths with treadmill belts moving at different speeds and found quicker adaptation in comparison to no feedback (Roemmich et al., 2016). By providing visual feedback of the errors, it simultaneously enhances voluntary correction and unconscious visuomotor remapping, resulting in increased adaptive behavior in response to a visual perturbation (Torres-Oviedo and Bastian, 2010; Roemmich et al., 2016; Statton et al., 2016). Sato et al. (2022) observed increased error with reward feedback during virtual split-belt walking in comparison to punishment and a no feedback group (Sato et al., 2022). Our findings partially agree with this study, in that reward feedback slows adaptation compared to supervised feedback. We further suggest that punishment is similarly detrimental to early adaptation and is like reward feedback. One possibility for this behavioral difference between the enhanced supervised and reinforcement feedback is the motor exploration of the task space. By providing graded reinforcement rather than binary (correct/incorrect), the Reward and Punishment groups may have explored different movements to optimize motor actions and thus limited monetary loss or increase monetary gains. However, by exploring the task space this slowed the rate of adaptation in comparison to the Supervised group. It is also important to highlight methodological differences between this study and Sato et al. (2022), specifically task used (step asymmetry) and the manner feedback was administered, which may affect the outcomes and interpretation of this study.

Interestingly this does contrast other previous work in the upper extremity where motor exploration increased the rate of adaptation, especially for Punishment (Song and Smiley-Oyen, 2017; Hill et al., 2020; Song et al., 2020). However, locomotor adaptation is complex as more task goals are present in comparison to upper extremity reaching. It should also be noted that all groups similarly adapted their movement pattern by the middle (Mid-Adaptation) and end of Adaptation (Adaptation Plateau), and the deficit is only during the Early Adaptation. This finding matches well with other studies in the locomotor and reaching domains, where adaptation to task demands can be achieved by leveraging sensory prediction (SPE) and reward prediction errors (RPE) to update to the sensory motor mapping over time.

Our results suggest that reward and punishment feedback impaired locomotor retention in comparison to their late adaptation performance. This result aligns well with a recent upper extremity reaching study demonstrating both reward and punishment feedback decreased performance during a similar errorless retention period (Huang et al., 2018; Hamel et al., 2021). Other studies have noted similar deficits, specifically with reward feedback, in the formation of motor memory (Steel et al., 2016, 2019; Van der Kooij et al., 2018). Our findings do contrast Hasson et al. (2015), where reward feedback demonstrated similar retention in comparison to a visual feedback group after a locomotor adaptation task (Hasson et al., 2015). These differences are expected since our learning paradigm is not identical to the one utilized by this study. Here we used a knee flexion task and numeric reinforcement in comparison to ankle inversion/eversion and categorical reinforcement, which may play a role in the differing findings.

These studies, along with others, suggest that motor adaptation and memory are mediated by different neural pathways. We propose the findings of this study that reinforcement utilizes different brain pathways and that the pathways used are task dependent. This idea would match the findings of Steel et al. (2016, 2019) where changes in functional connectivity were different depending on the task and type of feedback received. In the current study’s case, reward and punishment may operate on different neural pathways that do not support the formation of robust motor memories of knee flexion angle task (Steel et al., 2016, 2019). It has also been found that generating an RPE during adaptation interferes with cerebellum’s ability to resolve SPE, thus reducing the implicit contribution to motor memory (Hamel et al., 2021). The current study provides credence to this claim given the known involvement of the cerebellum during locomotor adaptation paradigms; thus, it is possible that during locomotor adaptation tasks, RPEs disrupt cerebellar processes leading to lower motor retention (Hinton et al., 2020; Sato and Choi, 2021). In summary, it can be inferred that the impact of punishments and rewards on motor memory is intricate, and their mere presence alone is insufficient to improve memory formation when performing the task in a new context.

Interestingly, the distribution among the errors during Retention for Reward and Punishment appears bimodal in nature, suggesting individual differences in the ability to retain the new walking pattern. This suggests the presence of reinforcement and non-reinforcement learners, within these groups. Previous research has demonstrated behavioral individual differences in reinforcement learning paradigms (Grunitzki et al., 2014; Aberg et al., 2016). Moreover, differences in neurophysiological responses have also been noted between reinforcement and non-reinforcement learners (Smillie et al., 2011; Kaiser et al., 2018; Le et al., 2024). In the case of this study, reinforcement learners (i.e., those who maintain their performance) may have a greater overall ability to integrate the abstracted numerical feedback and may utilize different neural mechanisms to modify motor behavior, thus resulting in better retention compared to others within their group. Though this study attempted to monitor this group level sensitivity to reinforcement with BAS/BIS scales, future studies in this domain should consider identifying these individual differences when implementing reinforcement to learning paradigms. Moreover, this underscores the need for future research to observe the neural correlates of reinforcement processing during locomotor adaptation. Thereby illuminating distinct neurophysiological patterns that can further differentiate reinforcement and non-reinforcement learners.

A large aftereffect is thought to be indicative of the amount of implicit adaptation achieved during the task and reflects a change in the internal representation of a movement. During Washout, all groups experienced an increase to their normal walking knee flexion angle in comparison to their baseline walking. This finding suggests that all groups were not simply relying on conscious adjustments to meet the task demands, instead some amount of adaptation experienced by the participants is implicit in nature. This agrees with a previous study using a similar knee flexion paradigm in healthy young adults (Statton et al., 2016), where participants displayed large aftereffects following an adaptation period and was the result of resolving the sensory prediction errors during the adaptation phase. Taken together, we propose that both reward and sensory prediction errors, generated by feedback received during adaptation, facilitate implicit locomotor adaptation similarly. This may be important to neurologically impaired populations, like persons with stroke, who previously demonstrated limited movement aftereffects following a knee flexion task with visually guided feedback (Cherry-Allen et al., 2018). In this case, reinforcement feedback may serve as an additional tool to leverage during similar interventions, but this is speculative and requires further investigation with this specific population.

To quantify the amount of task savings, all groups reengaged the knee flexion task with feedback restored following Washout. We found no difference between the groups during the Readaptation phase, and all groups displayed faster readaptation to the task compared to their previous exposure. Previous studies in the upper extremity demonstrate mixed results where reward and punishment similarly enhance savings (Quattrocchi et al., 2017) or have no differences with reward feedback compared to a neutral feedback control (Palidis et al., 2021). Punishment feedback was found to increase locomotor savings of a split belt walking task, while no feedback and reward failed to induce a robust pattern of locomotor savings (Sato et al., 2022). The authors posited that punishment induced more value to the newly learned pattern, resulting in more savings. We were not able to replicate these findings in our task, which may be related to differences in the task utilized (step asymmetry vs. knee flexion adaptation). Our results most closely resemble those of Leech and Roemmich (2018), who demonstrated that despite the type of feedback received during initial adaptation, they resulted in similar levels of savings (Leech and Roemmich, 2018). Taken together with our findings, we propose that the mechanisms governing savings are independent from adaptation and that reinforcement does not impact locomotor task savings. Alternatively, it is possible that the groups were able to more quickly implement a previously used strategy that was bolstered by changes to the task sensorimotor mapping to achieve a faster adaptation. However, this is speculative as we did not measure the usage of an explicit strategy during Readaptation.

The findings of this study may hold significance in understanding the potential outcomes of rehabilitation. Specifically, the current findings suggest using supervised feedback can enhance memory and acquisition of a joint-angle adaptation task. In scenarios where disease symptoms affect control of knee movement, such as stiff-knee gait (common in chronic stroke patients), enhanced supervised feedback could be utilized to modify knee flexion movements. However, in a situation where sensory feedback is attenuated by disease, reinforcement may be used to supplement motor performance. Reinforcement could be used to help with certain aspects of memory, such as savings.

## Limitations and future directions

Although this work provides valuable insight into the underlying learning processes occurring during a locomotor adaptation task with reward and punishment, it is not without limitations. We did not test if participants were utilizing an explicit strategy during the locomotor task which has been shown to impact behavioral performance (Codol et al., 2020). Though the sample size is comparable to previous studies in this domain (Hasson et al., 2015; Sato et al., 2022), studies of this nature benefit from larger sample sizes and this study has a limited sample size (*n* = 33). Due to limitations in equipment, we were unable to examine the kinematics of the hip during the locomotor adaptation task, which changed in compensation to the new knee flexion pattern (Statton et al., 2016; Cherry-Allen et al., 2018). While we believe this study has implications for neurological disease and rehabilitation, it was nevertheless conducted with neurologically intact, healthy young adults, which limits generalizability of its findings to aged and clinical populations.

Future studies should integrate measures of brain and muscle activity to provide a better understanding of the neural processes underlying these changes in behavior seen with supervised and reinforcement feedback during locomotion. This study focused on the short-term effects of reinforcement feedback, leaving the long-term difference as a subject of future experiments. Moreover, previous upper extremity studies have shown that reinforcement works differently in older adults and persons with stroke, thus providing an additional avenue for study in population with potentially less sensory feedback modulation (Quattrocchi et al., 2017; Huang et al., 2018).

## Conclusion

In conclusion, we demonstrate effects of reinforcement and enhanced sensory feedback on various aspects of locomotor adaptation and retention. Specifically, we demonstrated the deficits of providing reinforcement (reward or punishment) during a locomotor adaptation task, as it decreases the rate of adaptation and impairs retention. However, despite the feedback type, engaging in the knee flexion adaptation induced a movement aftereffect and task savings, suggesting changes to the neuromotor plasticity. Our results expand on previous studies by proposing the effects of reinforcement may be highly task dependent and a comparison of upper and lower extremity adaptation should be done with caution.

## Data availability statement

The raw data supporting the conclusions of this article will be made available by the authors, without undue reservation.

## Ethics statement

The studies involving humans were approved by Northern Illinois University Institutional Review Board. The studies were conducted in accordance with the local legislation and institutional requirements. The participants provided their written informed consent to participate in this study.

## Author contributions

CH: Conceptualization, Data curation, Formal analysis, Investigation, Methodology, Project administration, Resources, Software, Supervision, Validation, Visualization, Writing – original draft, Writing – review & editing. ES: Conceptualization, Methodology, Resources, Writing – original draft, Writing – review & editing. LB: Data curation, Investigation, Writing – original draft, Writing – review & editing. MW: Investigation, Methodology, Writing – original draft, Writing – review & editing, Data curation, Formal analysis. TW: Investigation, Methodology, Writing – original draft, Writing – review & editing, Conceptualization.

## References

[ref1] Aberg K. C. Doell K. C. Schwartz S. (2016). Linking individual learning styles to approach-avoidance motivational traits and computational aspects of reinforcement learning. PLoS One 11:e0166675. doi: 10.1371/journal.pone.0166675, PMID: 27851807 PMC5113060

[ref2] Aluja A. Blanch A. (2011). Neuropsychological behavioral inhibition system (BIS) and behavioral approach system (BAS) assessment: a shortened sensitivity to punishment and sensitivity to reward questionnaire version (SPSRQ-20). J. Pers. Assess. 93, 628–636. doi: 10.1080/00223891.2011.608760, PMID: 21999386

[ref3] Bakkum A. Donelan J. M. Marigold D. S. (2020). Challenging balance during sensorimotor adaptation increases generalization. J. Neurophysiol. 123, 1342–1354. doi: 10.1152/jn.00687.2019, PMID: 32130079 PMC7191513

[ref4] Bakkum A. Donelan J. M. Marigold D. S. (2021). Savings in sensorimotor learning during balance-challenged walking but not reaching. J. Neurophysiol. 125, 2384–2396. doi: 10.1152/jn.00627.2020, PMID: 34038257

[ref5] Baranek R., Inertial measurement unit - data fusion and visualization using MATLAB. IFAC proceedings volumes (IFAC-papers online). Elsevier; (2012). 12–16, 45

[ref6] Baumann O. Borra R. J. Bower J. M. Cullen K. E. Habas C. Ivry R. B.. (2015). Consensus paper: the role of the cerebellum in perceptual processes. Cerebellum 14, 197–220. doi: 10.1007/s12311-014-0627-7, PMID: 25479821 PMC4346664

[ref7] Boisgontier M. P. Cheval B. (2016). The anova to mixed model transition. Neurosci. Biobehav. Rev. 68, 1004–1005. doi: 10.1016/j.neubiorev.2016.05.034 27241200

[ref8] Buurke T. J. W. Sharma N. Swart S. B. van der Woude L. H. V. den Otter R. Lamoth C. J. C. (2022). Split-belt walking: an experience that is hard to forget. Gait Posture 97, 184–187. doi: 10.1016/j.gaitpost.2022.08.003, PMID: 35986959

[ref9] Cashaback J. G. A. Lao C. K. Palidis D. J. Coltman S. K. McGregor H. R. Gribble P. L. (2019). The gradient of the reinforcement landscape influences sensorimotor learning. PLoS Comput. Biol. 15:e1006839. doi: 10.1371/journal.pcbi.1006839, PMID: 30830902 PMC6417747

[ref10] Cherry-Allen K. M. Statton M. A. Celnik P. A. Bastian A. J. (2018). A dual-learning paradigm simultaneously improves multiple features of gait post-stroke. Neurorehabil. Neural Repair 32, 810–820. doi: 10.1177/1545968318792623, PMID: 30086670 PMC6143413

[ref11] Choi J. T. Jensen P. Nielsen J. B. (2016). Locomotor sequence learning in visually guided walking. J. Neurophysiol. 115, 2014–2020. doi: 10.1152/jn.00938.2015, PMID: 26864768 PMC4869504

[ref12] Codol O. Holland P. J. Manohar S. G. Galea J. M. (2020). Reward-based improvements in motor control are driven by multiple error-reducing mechanisms. J. Neurosci. 40, 3604–3620. doi: 10.1523/JNEUROSCI.2646-19.2020, PMID: 32234779 PMC7189755

[ref13] Diederen K. M. J. Ziauddeen H. Vestergaard M. D. Spencer T. Schultz W. Fletcher P. C. (2017). Dopamine modulates adaptive prediction error coding in the human midbrain and striatum. J. Neurosci. 37, 1708–1720. doi: 10.1523/JNEUROSCI.1979-16.2016, PMID: 28202786 PMC5320604

[ref14] Doan Q. V. Pham D. D. (2019). Inertial navigation algorithm for trajectory of front-wheel walker estimation. Heliyon. 5:e01896. doi: 10.1016/j.heliyon.2019.e01896, PMID: 31338448 PMC6579904

[ref15] Dzewaltowski A. C. Hedrick E. A. Leutzinger T. J. Remski L. E. Rosen A. B. (2021). The effect of Split-Belt treadmill interventions on step length asymmetry in individuals Poststroke: a systematic review with meta-analysis. Neurorehabil. Neural. Repair 35, 563–575. doi: 10.1177/15459683211011226 33978525

[ref16] Galea J. M. Mallia E. Rothwell J. Diedrichsen J. (2015). The dissociable effects of punishment and reward on motor learning. Nat. Neurosci. 18, 597–602. doi: 10.1038/nn.3956, PMID: 25706473

[ref17] Galea J. M. Vazquez A. Pasricha N. Orban De Xivry J. J. Celnik P. (2011). Dissociating the roles of the cerebellum and motor cortex during adaptive learning: the motor cortex retains what the cerebellum learns. Cereb. Cortex 21, 1761–1770. doi: 10.1093/cercor/bhq246, PMID: 21139077 PMC3138512

[ref18] Giboin L. S. Tokuno C. Kramer A. Henry M. Gruber M. (2020). Motor learning induces time-dependent plasticity that is observable at the spinal cord level. J. Physiol. 598, 1943–1963. doi: 10.1113/JP278890, PMID: 32115702

[ref19] Gonzalez-Rubio M. Velasquez N. F. Torres-Oviedo G. (2019). Explicit control of step timing during split-belt walking reveals interdependent recalibration of movements in space and time. Front. Hum. Neurosci. 13, 1–12. doi: 10.3389/fnhum.2019.00207 31333429 PMC6619396

[ref20] Grunitzki R De Ramos GO Bazzan ALC. Individual versus difference rewards on reinforcement learning for route choice. Proc. 2014 Brazilian Conf. Intell. Syst. BRACIS, (2014). 2014; 253–258

[ref21] Hamel R. La Fontaine D. Lepage J. F. Bernier P. M. (2021). Punishments and rewards both modestly impair visuomotor memory retention. Neurobiol. Learn. Mem. 185:107532. doi: 10.1016/j.nlm.2021.107532, PMID: 34592470

[ref22] Hamel R. Savoie F. A. Lacroix A. Whittingstall K. Trempe M. Bernier P. M. (2018). Added value of money on motor performance feedback: increased left central beta-band power for rewards and fronto-central theta-band power for punishments. NeuroImage 179, 63–78. doi: 10.1016/j.neuroimage.2018.06.032, PMID: 29894825

[ref23] Hasson C. J. Manczurowsky J. Yen S. C. (2015). A reinforcement learning approach to gait training improves retention. Front. Hum. Neurosci. 9:459. doi: 10.3389/fnhum.2015.00459 26379524 PMC4550775

[ref24] Hester R. Murphy K. Brown F. L. Skilleter A. J. (2010). Punishing an error improves learning: the influence of punishment magnitude on error-related neural activity and subsequent learning. J. Neurosci. 30, 15600–15607. doi: 10.1523/JNEUROSCI.2565-10.2010, PMID: 21084615 PMC6633683

[ref25] Hill C. M. Stringer M. Waddell D. E. Del Arco A. (2020). Punishment feedback impairs memory and changes cortical feedback-related potentials during motor learning. Front. Hum. Neurosci. 14:294. doi: 10.3389/fnhum.2020.00294, PMID: 32848669 PMC7419689

[ref26] Hill C. M. Waddell D. E. Del Arco A. (2021). Cortical preparatory activity during motor learning reflects visuomotor retention deficits after punishment feedback. Exp. Brain Res. 239, 3243–3254. doi: 10.1007/s00221-021-06200-x, PMID: 34453554

[ref27] Hinton D. C. Conradsson D. M. Paquette C. (2020). Understanding human neural control of short-term gait adaptation to the Split-belt treadmill. Neuroscience 451, 36–50. doi: 10.1016/j.neuroscience.2020.09.055, PMID: 33039522

[ref28] Hoogkamer W. Bruijn S. M. Sunaert S. Swinnen S. P. Van Calenbergh F. Duysens J. (2015). Adaptation and aftereffects of split-belt walking in cerebellar lesion patients. J. Neurophysiol. 114, 1693–1704. doi: 10.1152/jn.00936.2014, PMID: 26203113 PMC4567611

[ref29] Huang J. Hegele M. Billino J. (2018). Motivational modulation of age-related effects on reaching adaptation. Front. Psychol. 9:413886. doi: 10.3389/fpsyg.2018.02285 PMC6255948 30515126

[ref30] Izawa J. Shadmehr R. (2011). Learning from sensory and reward prediction errors during motor adaptation. PLoS Comput. Biol. 7:e1002012. doi: 10.1371/journal.pcbi.1002012, PMID: 21423711 PMC3053313

[ref31] Jayaram G. Tang B. Pallegadda R. Vasudevan E. V. L. Celnik P. Bastian A. (2012). Modulating locomotor adaptation with cerebellar stimulation. J. Neurophysiol. 107, 2950–2957. doi: 10.1152/jn.00645.2011, PMID: 22378177 PMC3378372

[ref32] Jocham G. Ullsperger M. (2009). Neuropharmacology of performance monitoring. Neurosci. Biobehav. Rev. 33, 48–60. doi: 10.1016/j.neubiorev.2008.08.011 18789964

[ref33] Kaiser R. H. Treadway M. T. Wooten D. W. Kumar P. Goer F. Murray L.. (2018). Frontostriatal and dopamine markers of individual differences in reinforcement learning: a multi-modal investigation. Cereb. Cortex 28, 4281–4290. doi: 10.1093/cercor/bhx281, PMID: 29121332 PMC6454484

[ref34] Krakauer J. W. Hadjiosif A. M. Xu J. Wong A. L. Haith A. M. (2019). Motor learning. Compr. Physiol. 9, 613–663. doi: 10.1002/cphy.c170043, PMID: 30873583

[ref35] Kumari N. Taylor D. Rashid U. Vandal A. C. Smith P. F. Signal N. (2020). Cerebellar transcranial direct current stimulation for learning a novel split-belt treadmill task: a randomised controlled trial. Sci Reports 101, 1–14. doi: 10.1038/s41598-020-68825-2 PMC7366632 32678285

[ref36] Le T. M. Oba T. Couch L. McInerney L. Li C. S. R. (2024). The neural correlates of individual differences in reinforcement learning during pain avoidance and reward seeking. eNeuro. 11:437. doi: 10.1523/ENEURO.0437-23.2024, PMID: 38365840 PMC10901196

[ref37] Leech K. A. Roemmich R. T. (2018). Independent voluntary correction and savings in locomotor learning. J. Exp. Biol. 221, 1–6. doi: 10.1038/s41598-017-18538-w PMC6104817 29903840

[ref38] Long A. W. Roemmich R. T. Bastian A. J. (2016). Blocking trial-by-trial error correction does not interfere with motor learning in human walking. J. Neurophysiol. 115, 2341–2348. doi: 10.1152/jn.00941.2015, PMID: 26912598 PMC4922458

[ref39] Malone L. A. Bastian A. J. Torres-Oviedo G. (2012). How does the motor system correct for errors in time and space during locomotor adaptation? J. Neurophysiol. 108, 672–683. doi: 10.1152/jn.00391.2011, PMID: 22514294 PMC4073916

[ref40] Morton S. M. Bastian A. J. (2006). Cerebellar contributions to locomotor adaptations during splitbelt treadmill walking. J. Neurosci. 26, 9107–9116. doi: 10.1523/JNEUROSCI.2622-06.2006, PMID: 16957067 PMC6674518

[ref41] Nikooyan A. A. Ahmed A. A. (2015). Reward feedback accelerates motor learning. J. Neurophysiol. 113, 633–646. doi: 10.1152/jn.00032.2014, PMID: 25355957

[ref42] Palidis D. J. McGregor H. R. Vo A. MacDonald P. A. Gribble P. L. (2021). Null effects of levodopa on reward-and error-based motor adaptation, savings, and anterograde interference. J. Neurophysiol. 126, 47–67. doi: 10.1152/jn.00696.2020, PMID: 34038228

[ref43] Quattrocchi G. Greenwood R. Rothwell J. C. Galea J. M. Bestmann S. (2017). Reward and punishment enhance motor adaptation in stroke. J. Neurol. Neurosurg. Psychiatry 88, 730–736. doi: 10.1136/jnnp-2016-314728, PMID: 28377451

[ref44] Quattrocchi G. Monaco J. Ho A. Irmen F. Strube W. Ruge D.. (2018). Pharmacological dopamine manipulation does not Alter reward-based improvements in memory retention during a Visuomotor adaptation task. eNeuro. 5, ENEURO.0453–ENEU17.2018. doi: 10.1523/ENEURO.0453-17.2018 30027109 PMC6051592

[ref45] Reisman D. S. Bastian A. J. Morton S. M. (2010). Neurophysiologic and rehabilitation insights from the split-belt and other locomotor adaptation paradigms. Phys. Ther. 90, 187–195. doi: 10.2522/ptj.20090073, PMID: 20023001 PMC2816031

[ref46] Reisman D. S. Block H. J. Bastian A. J. (2005). Interlimb coordination during locomotion: what can be adapted and stored? J. Neurophysiol. 94, 2403–2415. doi: 10.1152/jn.00089.2005, PMID: 15958603

[ref47] Roemmich R. T. Bastian A. J. (2015). Two ways to save a newly learned motor pattern. J. Neurophysiol. 113, 3519–3530. doi: 10.1152/jn.00965.2014, PMID: 25855699 PMC4461882

[ref48] Roemmich R. T. Bastian A. J. (2018). Closing the loop: from motor neuroscience to neurorehabilitation. Annu. Rev. Neurosci. 41, 415–429. doi: 10.1146/annurev-neuro-080317-062245 29709206

[ref49] Roemmich R. T. Long A. W. Bastian A. J. (2016). Seeing the errors you feel enhances locomotor performance but not learning. Curr. Biol. 26, 2707–2716. doi: 10.1016/j.cub.2016.08.012, PMID: 27666970 PMC5081226

[ref50] Sato S. Choi J. T. (2021). Neural control of human locomotor adaptation: lessons about changes with aging. Neuroscientist 28, 469–484. doi: 10.1177/10738584211013723 34014124

[ref51] Sato S. Cui A. Choi J. T. (2022). Visuomotor errors drive step length and step time adaptation during ‘virtual’ split-belt walking: the effects of reinforcement feedback. Exp. Brain Res. 240, 511–523. doi: 10.1007/s00221-021-06275-6, PMID: 34816293

[ref52] Severini G Zych M. Locomotor adaptations: paradigms, principles and perspectives. 4, Progress in biomedical engineering. IOP Publishing; (2022). 42003. doi: 10.1088/2516-1091/ac91b6

[ref53] Shadmehr R. Smith M. A. Krakauer J. W. (2010). Error correction, sensory prediction, and adaptation in motor control. Annu. Rev. Neurosci. 33, 89–108. doi: 10.1146/annurev-neuro-060909-153135 20367317

[ref54] Smillie L. D. Cooper A. J. Pickering A. D. (2011). Individual differences in reward–prediction-error: extraversion and feedback-related negativity. Soc. Cogn. Affect. Neurosci. 6, 646–652. doi: 10.1093/scan/nsq078, PMID: 20855297 PMC3190205

[ref55] Song Y. Lu S. Smiley-Oyen A. L. (2020). Differential motor learning via reward and punishment. Q. J. Exp. Psychol. 73, 249–259. doi: 10.1177/1747021819871173, PMID: 31382855

[ref56] Song Y. Smiley-Oyen A. L. (2017). Probability differently modulating the effects of reward and punishment on visuomotor adaptation. Exp. Brain Res. 235, 3605–3618. doi: 10.1007/s00221-017-5082-5, PMID: 28887626

[ref57] Spampinato D. Celnik P. (2021). Multiple motor learning processes in humans: defining their neurophysiological bases. Neuroscientist 27, 246–267. doi: 10.1177/1073858420939552, PMID: 32713291 PMC8151555

[ref58] Spampinato D. A. Satar Z. Rothwell J. C. (2019). Combining reward and M1 transcranial direct current stimulation enhances the retention of newly learnt sensorimotor mappings. Brain Stimul. 12, 1205–1212. doi: 10.1016/j.brs.2019.05.015, PMID: 31133478 PMC6709642

[ref59] Statton M. A. Toliver A. Bastian A. J. (2016). A dual-learning paradigm can simultaneously train multiple characteristics of walking. J. Neurophysiol. 115, 2692–2700. doi: 10.1152/jn.00090.2016, PMID: 26961100 PMC4922483

[ref60] Steel A. Silson E. H. Stagg C. J. Baker C. I. (2016). The impact of reward and punishment on skill learning depends on task demands. Sci. Rep. 6:36056. doi: 10.1038/srep36056 27786302 PMC5081526

[ref61] Steel A. Silson E. H. Stagg C. J. Baker C. I. (2019). Differential impact of reward and punishment on functional connectivity after skill learning. NeuroImage 189, 95–105. doi: 10.1016/j.neuroimage.2019.01.009, PMID: 30630080 PMC7612345

[ref62] Torres-Oviedo G. Bastian A. J. (2010). Seeing is believing: effects of visual contextual cues on learning and transfer of locomotor adaptation. J. Neurosci. 30, 17015–17022. doi: 10.1523/JNEUROSCI.4205-10.2010, PMID: 21159971 PMC3025449

[ref63] Torres-Oviedo G Vasudevan E Malone L Bastian AJ. Locomotor adaptation. Progress in brain research. Prog Brain Res; (2011). 65–74 10.1016/B978-0-444-53752-2.00013-8 PMC3738197 21741544

[ref64] Van der Kooij K. Wijdenes L. O. Rigterink T. Overvliet K. E. Smeets J. B. J. (2018). Reward abundance interferes with error-based learning in a visuomotor adaptation task. PLoS One 13:e0193002. doi: 10.1371/journal.pone.0193002, PMID: 29513681 PMC5841744

[ref65] Wei K. Körding K. (2009). Relevance of error: what drives motor adaptation? J. Neurophysiol. 101, 655–664. doi: 10.1152/jn.90545.2008, PMID: 19019979 PMC2657056

[ref66] Wrase J. Kahnt T. Schlagenhauf F. Beck A. Cohen M. X. Knutson B.. (2007). Different neural systems adjust motor behavior in response to reward and punishment. NeuroImage 36, 1253–1262. doi: 10.1016/j.neuroimage.2007.04.001, PMID: 17521924

[ref67] Wu Y. Morita M. Izawa J. (2022). Reward prediction errors, not sensory prediction errors, play a major role in model selection in human reinforcement learning. Neural Netw. 154, 109–121. doi: 10.1016/j.neunet.2022.07.002 35872516

[ref68] Yin C. I. Gao T. Li B. (2023). The effect of combining punishment and reward can transfer to opposite motor learning. PLoS One 18:282028. doi: 10.1371/journal.pone.0282028 PMC10085010 37036847

